# NG2-Glia Transiently Overcome Their Homeostatic Network and Contribute to Wound Closure After Brain Injury

**DOI:** 10.3389/fcell.2021.662056

**Published:** 2021-04-27

**Authors:** Axel von Streitberg, Sarah Jäkel, Jaime Eugenin von Bernhardi, Christoph Straube, Felix Buggenthin, Carsten Marr, Leda Dimou

**Affiliations:** ^1^Physiological Genomics, Biomedical Center, Ludwig-Maximilians-University Munich, Munich, Germany; ^2^Molecular and Translational Neuroscience, Department of Neurology, Ulm University, Ulm, Germany; ^3^Institute of Computational Biology, Helmholtz Zentrum München, Neuherberg, Germany

**Keywords:** proliferation, migration, polarization, stab wound injury, *in vivo* two-photon imaging

## Abstract

In the adult brain, NG2-glia represent a cell population that responds to injury. To further investigate if, how and why NG2-glia are recruited to the injury site, we analyzed in detail the long-term reaction of NG2-glia after a lesion by time-lapse two-photon *in vivo* microscopy. Live imaging over several weeks of GFP-labeled NG2-glia in the stab wounded cerebral cortex revealed their fast and heterogeneous reaction, including proliferation, migration, polarization, hypertrophy, or a mixed response, while a small subset of cells remained unresponsive. At the peak of the reaction, 2–4 days after the injury, NG2-glia accumulated around and within the lesion core, overcoming the homeostatic control of their density, which normalized back to physiological conditions only 4 weeks after the insult. Genetic ablation of proliferating NG2-glia demonstrated that this accumulation contributed beneficially to wound closure. Thus, NG2-glia show a fast response to traumatic brain injury (TBI) and participate in tissue repair.

## Introduction

Traumatic brain injury (TBI) involves sudden nerve tissue damage caused by an external mechanical force to the head. TBI is the primary cause of death, especially in young adults, and significantly increases the risk of long-term disability for survivors, representing substantial socio-economic challenges at a global level (Roozenbeek et al., 2013). The key reason for this is the limited recovery capacity of the brain, which results in poor treatment outcomes for insults to the central nervous system (CNS). Despite the fact that this situation has been heavily investigated for decades, there are still prominent gaps in our knowledge regarding the events taking place after a brain injury. Glial cells are major players for tissue regeneration and they react strongly to brain insults using a diverse spectrum of cellular processes, such as changes in morphology (like hypertrophy and polarization), migration, proliferation, and, in some cases, differentiation. It is therefore vitally important for future clinical research, in order to improve clinical outcomes, to better understand the alterations in glial populations and their contributions to tissue recovery.

The reaction and role of some glial cell populations have been extensively described following mechanical traumas. For instance, cortical astrocytes overexpress GFAP after brain insult (Sofroniew and Vinters, 2010) but do not migrate into the injury site, while only a limited number of astrocytes divide and polarize toward the lesion (Sofroniew and Vinters, 2010; Bardehle et al., 2013). Astrocytes participate in scar formation, inflammation modulation, damage control, and blood–brain barrier (BBB) repair, among other functions (Sofroniew and Vinters, 2010). Likewise, microglia get activated, extend processes toward, and begin to migrate into the lesion area where they proliferate, phagocytose cell debris, and form a scar within a few hours (Kettenmann et al., 2011). Nonetheless, the cellular responses and functions of other cell types after injury—such as NG2-glia—are less known.

NG2-glia, also known as oligodendrocyte progenitor cells, represent 5–10% of the total cell population and generate oligodendrocytes in the developing and adult CNS (Dimou et al., 2008; Nishiyama et al., 2009). Additionally, they are the only proliferating cells in the healthy adult cerebral cortex, and are organized in non-overlapping domains all over the brain and spinal cord (Nishiyama et al., 2009; Simon et al., 2011). Under physiological conditions, the distribution and homeostatic control of their population density are achieved by a self-repulsion mechanism, which regulates the proliferation and short-range migration of these cells after cell death or differentiation (Hughes et al., 2013). This observation raises the question: why do NG2-glia retain a constant population size, even after developmental myelination completion? Their cellular distribution suggests that they may have other functions besides oligodendrogenesis. Indeed, after TBI, as well as in models of Alzheimer’s disease (AD; Behrendt et al., 2013), demyelination (Di Bello et al., 1999; Levine and Reynolds, 1999), and other neurodegenerative diseases (Kang et al., 2013), NG2-glia are, together with residential microglia, the first cells to respond with a rapid and transient reaction (Simon et al., 2011).

Some of the NG2-glia responses involve changes in their morphology and upregulation of the proteoglycan NG2 as well as an increase in their cell number by shortening their cell cycle length and the recruitment of quiescent NG2-glia into the cell cycle (Simon et al., 2011). Indeed, the roles that the reaction of NG2-glia to brain injury plays are not well known. It is speculated that the accumulation of NG2-glia in the scar contributes to the inhibition of axonal growth (Chen et al., 2002) and, indeed, inhibiting the proliferation of NG2-glia with the antimitotic drug Cytarabine (AraC), which also affects microglia, leads to enhanced axonal regeneration (Rhodes et al., 2003). Nevertheless, it has been shown that regenerative axons preferentially elongate on vimentin-positive NG2-glia after spinal cord injury (Busch et al., 2010).

Most information surrounding the response of NG2-glia to trauma has been obtained from analysis of single time points of post-mortem tissue, and there are only few full live imaging experiments showing single cell dynamics in response to injury. From the latter, it has been shown that toxin-induced demyelination of *ex vivo* postnatal forebrain slices leads to the acceleration of NG2-glia differentiation after division (Hill et al., 2014). Furthermore, after a focal laser lesion of single cells in the cortex parenchyma, juxtaposed progenitors react relatively homogenously with initial migration followed by proliferation toward the insult (Hughes et al., 2013). Despite those findings, there are important concerns regarding the interpretation and translational accuracy of these data in the context of brain trauma. First, none of the types of injury studied properly follows the technical definition of TBI. Second, laser-induced lesion produces an injury smaller than 25 μm in diameter, and the cauterizing nature of heat-based methodologies may lead to a minimal impact on BBB integrity. Moreover, both factors might reduce the earlier contribution of peripheral immune cells and systemic signals, restricting the response of NG2-glia only close to the insult. Likewise, the white matter toxin-based lesion in cultured brain slices disregards the exchange between cells of the wounded brain and the periphery. Third, the analysis on both pieces of research has been only carried out at the lesion core or its immediate surrounding (not further than 75 μm away), which negates the effect of cells further apart. Fourth, it is not clear whether all NG2-glia have this stereotypical behavior or whether only a fraction of them have the capacity to react to the insult. Consequently, many questions regarding the response of NG2-glia after a more extensive TBI in the adult cerebral cortex are still unresolved. Key questions remain: what are the dynamics of individual cells over time? Does the injury size affect NG2-glia activation? Is the proliferative and migratory response of NG2-glia restricted to the replacement of depleted NG2-glia, or do the cells accumulate due to a breakdown of their cellular homeostasis? How long are NG2-glia responsive to a mechanical insult and do they show a homogenous behavior among the population? And most importantly, what is the function of these reactive NG2-glia?

To answer all these questions, we employed *in vivo* two-photon live imaging to follow NG2-glia after different sizes of stab wound injury (SWI) over time. SWI has several advantages over other TBI models as it can be adjusted to produce different injury sizes with low variability among experiments. Additionally, it reduces the amount of bleeding that can intervene with the optics of the microscope. Here, we were able to observe a fast, robust, and heterogeneous reaction of NG2-glia within the direct vicinity of the lesion, observable as early as 2 days after injury. NG2-glia migrated rapidly into the injury site, together with proliferation, leading to a strong increase in their number, illustrating a breakdown of their homeostatic control. This sharp increase in NG2-glia was resolved after 4 weeks, and their morphology and distribution returned to a level comparable to physiological conditions. Finally, genetic ablation of proliferating NG2-glia leading to decreased cell numbers after injury resulted in a delayed wound closure, suggesting a crucial role of NG2-glia and their reaction in the process of nerve tissue healing.

## Materials and Methods

### Animals

Male and female adult (3–4 months old) Sox10iCreER^*T*2^xCAG-eGFP (Simon et al., 2012) and Sox10iCreER^*T*2^xCAG-eGFPxEsco2^*fl/fl*^ (Whelan et al., 2012; Schneider et al., 2016) mice received three times every second day 0.4 mg tamoxifen per gram of body weight by oral gavage for a week every second day (stock solution: 40 mg/ml tamoxifen in corn oil with 10% EtOH). On some occasions, a reduced amount (one-time gavaging; 0.4 μg tamoxifen per gram of body weight) was used to label fewer cells in the Sox10iCreER^*T*2^xCAG-eGFP line. At least 9 days after recombination, a cranial window was introduced on the animals’ skull and, thereafter, mice were anesthetized by intraperitoneal injection of midazolam (5 mg/kg of body weight), medetomidine (0.5 mg/kg), and fentanyl (0.05 mg/kg). After anesthesia, a unilateral craniotomy was performed using a high-speed dental drill on the skull above the somatosensory cortex followed by a small punctate (depth of ∼0.7 mm and length of ∼0.1 mm) or an extensive SWI (depth of ∼0.7 mm and length of ∼1 mm) using a 19 lancet-shaped gauge knife. For the cranial window, a glass coverslip (5 mm diameter) was fixed over the craniotomy and sealed with dental acrylic (Paladur, Heraeus). For some of the longer imaging time points after injury [4 days post-injury (dpi)], the craniotomy and injury were performed as follows. Instead of sealing with a cranial window, the removed skull piece was placed on the craniotomy and sutured. Four days later, the skull piece was removed and the craniotomy was sealed with a cranial window as described above. For the control group, a craniotomy followed by the placement of a cranial window was performed as described above without any injury. Afterward, a metal head bar was placed on the other hemisphere to allow head fixation during imaging and 50 μl of a Texas-Red-conjugated dextran solution (70 kDa; Molecular Probes D1864, 10 mg/ml in 0.9% NaCl) was intravenously injected (ventral caudal artery) to label the blood vessels. After surgery and imaging, antagonization of the anesthesia was induced via injection of atipamezole (2.5 mg/kg), flumazenil (0.5 mg/kg), and naloxone (1.2 mg/kg). All experiments were performed in accordance and under the Guidelines of “Use of Animals and Humans in Neuroscience Research” revised and approved by the Society of Neuroscience, and licensed by the State of Upper Bavaria.

### *In vivo* Two-Photon Microscopy

Anesthetized animals were kept in position via a head bar on a custom made, heated stereotactic stage, orientated perpendicular to the optical axis of the microscope, and imaging was performed with an Olympus FV 1000MPE microscope equipped with a multi-photon, near infrared, pulsed MaiTai HP DeepSee laser (Spectra Physics), a 20x water immersion objective (1.0 NA), an FV10-MROPT Filter (BA = 420–500 nm for detection of second harmonic signals; BA = 515–560 nm for detection of GFP; BA = 590–650 for detection of Texas-Red), and internal photomultiplier tube detectors. The laser was tuned to 910 nm, and the laser intensity was adjusted accordingly to tissue depth (<50 mW). Emission of green fluorescence of intrinsic eGFP expression of recombined Sox10 expressing cells, red fluorescence of Texas-red labeled blood vessels, and blue second harmonic signal (detectable at half the emission wavelength ∼460 nm) were detected and optical sections with the resolution of 1024 × 1024 in the *x*–*y* dimension were recorded every 2 μm to a depth of maximal 500 μm below the dura. The orientation of the image plain was controlled by scanning the dura mater before each imaging session (Scheibe et al., 2011). To re-identify and re-image the area of interest at later time points, the labeled blood vessels and the stable oligodendrocytes were used as landmarks. The first imaging session was performed immediately after surgery (0 dpi; ∼30 min after the procedure), and imaging was repeated on days 2, 4, 6, 8, 11, 21, and 28.

### Image Processing and Analysis

**FIGURE 1:**
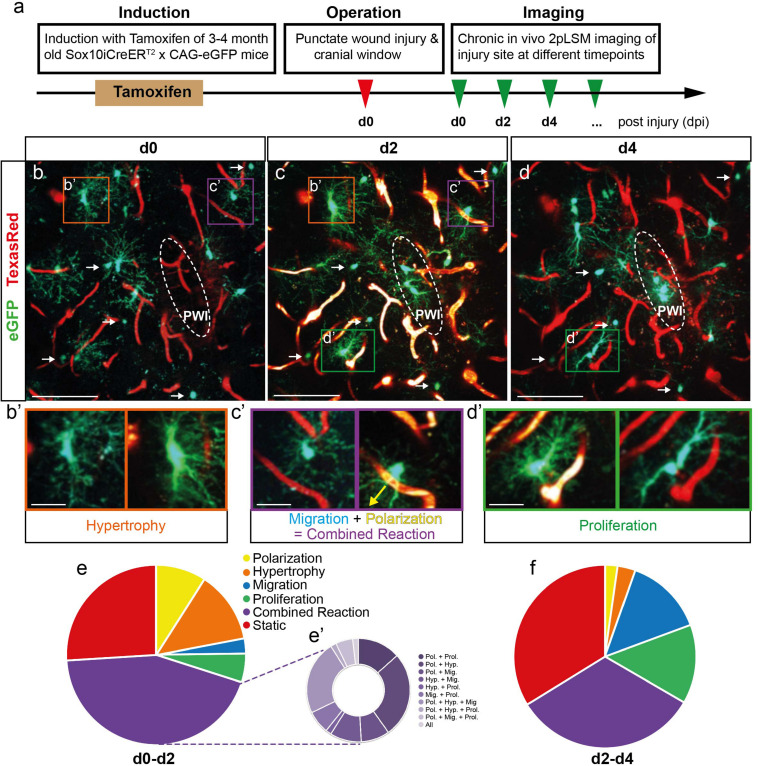
Fast and heterogeneous reaction of NG2-glia after injury. **(a)** Schematic illustration of the experimental procedure. **(b–d)** Images of GFP^+^ NG2-glia and oligodendrocytes (white arrows) surrounding a punctate wound injury (PWI; white dashed ellipse) at d0, d2, and d4 after the injury. Blood vessels are labeled with Texas-Red dextran (red). **(b’–d’)** Examples of cells (higher magnification from **b–d**) showing hypertrophy **(b’)**, the combined reaction of migration and polarization toward the injury (**c’**; yellow arrow indicates the direction towards the injury) and proliferation **(d’)**. **(e–f)** Pie charts represent the heterogeneous reaction of all NG2-glia surrounding the injury site between 0 and 2dpi (**e**; Polarization represents the cells polarizing toward the injury; the classification of the multiple reactions is represented in pie **e’**) and 2 and 4 dpi (**f**; n = 220 cells from 8 animals for d0–d2 and n = 180 cells from 6 animals for d2–d4). Images show maximum intensity projections of 30μm deep stacks. Scale bars represent 100μm in **b–d** and 25μm in **b’–d’**.

**FIGURE 2:**
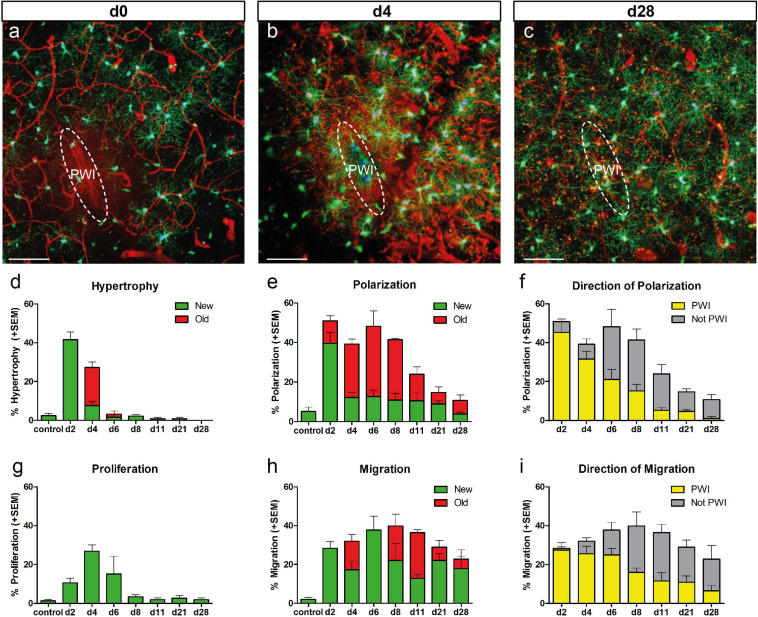
Temporal reaction of NG2-glia after injury. **(a–c)** Images of NG2-glia around the injury site at d0 **(a)**, d4 **(b)**, and d28 **(c)** after PWI. **(d,e,g,h)** Graphs depict the percentage (mean + SEM) of cells showing hypertrophy, polarization, proliferation and migration at the given timepoints (n = 3 − 8 animals per timepoint). “New” (green bars) represent the cells showing hypertrophy **(d)**, polarization **(e)**, proliferation **(g)** and migration **(h)** for the first time at the indicated timepoint. “Old” (red bars) represent cells which showed this behavior already at the previous timepoint. **(f,i)** Directionality of polarized **(f)** or migrating **(i)** cells (mean + SEM; yellow bars represent the percentage of polarized cells with a direction towards the quadrant enclosing the PWI; grey bars represent percentage of polarized cells towards the remaining 3 quadrants) over time. n = 8 mice for timepoint d2, n = 6 mice for timepoint d4, n = 4 mice for timepoint d8, n = 3 mice for all other timepoints; mostly 20–30 cells per animal). Images show maximum intensity projections of 30μm deep stacks. Scale bars represent 100μm.

**FIGURE 3:**
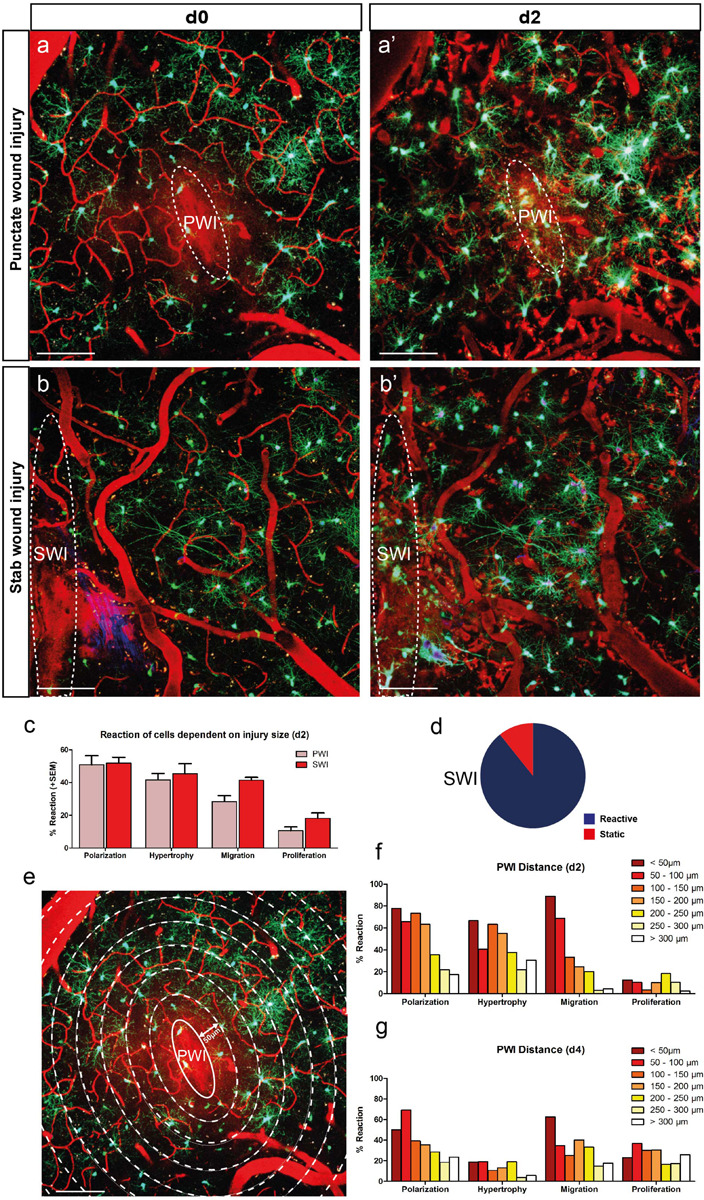
The degree of NG2-glia reaction depends on the size and proximity to the injury. **(a,b)** Images of the NG2-glia reaction between d0 and d2 after PWI **(a,a’)** and the bigger stab wound injury (SWI) **(b,b’)**. **(C)** NG2-glia show a stronger reaction after SWI compared to PWI (mean + SEM; n = 8 mice for PWI and n = 3 mice for SWI) with a lower percentage of static cells at 2dpi (d; compare to Fig. 1e). **(e–g)** Cells in closer proximity to the injury show increased reactivity compared to the ones further away from the lesion core at d2 **(e,f)**. This difference was less pronounced at 4dpi (g, polarization represents cells directed toward the injury; n = 220 cells from 8 animals at d2 and n = 180 cells from 6 animals at d4). Images show maximum intensity projections of 30μm deep stacks. Scale bars represent 100μm.

**FIGURE 4:**
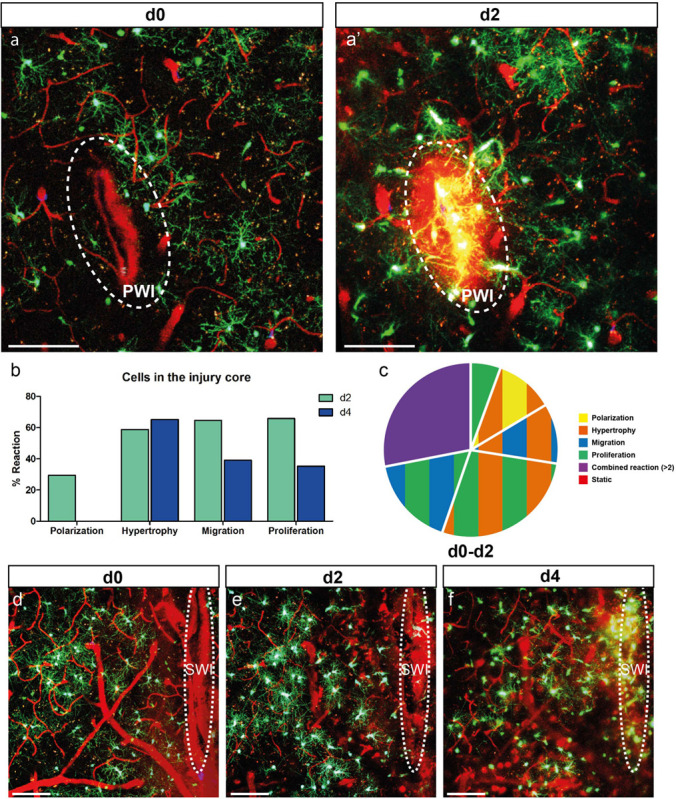
NG2-glia fill the injury core. **(a)** Images of NG2-glia at d0 **(a)** and d2 **(a’)** after PWI. Dotted circle indicates the core of the injury that corresponds to the analyzed area. **(b)** Graph showing a strong reactivity of these cells for all criteria (except polarization) at d2 and d4 after injury. **(c)** Pie chart of the heterogeneous reaction between 0 and 2dpi of NG2-glia showing no static cells (Polarization represents cells directed toward the injury; n = 34 cells from 7 animals for d2 and n = 23 cells from 4 animals at d4). Images show maximal projections of 20μm deep stacks. Scale bars represent 100μm. **(d–f)** Images of 0, 2 and 4 days after SWI showing NG2-glia only filling up the injury core at 4dpi. White ellipse represents the injury site. Images show maximum intensity projections of 30μm deep stacks. Scale bars represent 100μm.

Recorded image stacks were processed and analyzed using Fiji (based on ImageJ 1.48i) software. To reduce background noise, stacks were smoothed slightly using Fiji’s two-dimensional Gaussian filter (sigma = 0.7–1.0) and, in some cases, the background was reduced using Fiji’s Subtract Background (radius = 50–500). Cells of interest were identified, and the channel showing the blood vessels was used to retrieve the cells at different time points. For each cell and time point, the approximate distance to the dura (visible due to the second harmonic signal in the blue channel) and the injury core was measured. Then, morphological characteristics were analyzed. A cell was considered polarized when most processes were orientated toward one direction, often combined with elongation of the cell body. The directionality of the polarization was assessed by subdividing the area surrounding the cell into four quadrants. The quadrant in which the lesion site was placed in the center was considered PWI direction and the remaining three as non-PWI direction. Cells were categorized according to their reaction in a given time point. For each group, percentages of the individual traits were calculated and compared to the other groups. To determine whether a response was new or old (Figure 2), the mother cell traits were counted for the two daughter cells as a preliminary reaction. For the reaction profiles (Figures 1e,f) and the distance analysis (Figures 3f,g) 254 cells from eight animals were pooled for d0–d2 (Figure 1e) and 222 cells from six animals for d2–d4 (Figure 1f). In addition, regarding the different reaction profiles (Figures 2d–i), 254 cells from eight animals and 222 cells from six animals were analyzed for 2 and 4 dpi, respectively. Furthermore, 144 cells from four animals (6 dpi), 148 cells from four animals (8 dpi), 115 cells from three animals (11 dpi), 151 cells from four animals (21 dpi), 110 cells from three animals (28 dpi), and 199 cells from three animals for the control were analyzed for the later time points. For the stab wound paradigm (Figures 3c,d), 121 cells from three animals were compared to the 254 cells (2 dpi; PWI). The analysis of the cells in the injury core (Figures 4b,c) includes 34 cells from seven animals (2 dpi) and 23 cells from four animals (4 dpi). The velocity and maximum migration assays (Supplementary Figures 5e,f) comprise 115 cells from three animals (14 dpi) and the cells used for Figures 2d–i. For the follow-up profiling (Supplementary Figures 2d, 3d, 5d, 6d) 157 cells from six animals were analyzed.

### Data Processing and Image Registration

To resolve linear shifts and morphological deformations in the image stacks, a two-step landmark based registration approach using Elastix v4.5 (Klein et al., 2010) was applied. First, the stacks of 0, 2, and 4 dpi were split into their three-color channels using Fiji (Supplementary Figure 4a) (Schindelin et al., 2012). Under the assumption that blood vessels do not change their spatial position over time, the blood vessel image stacks were used as landmarks for registration; 0 dpi was defined as the fixed image stack and 2 and 4 dpi as the “moving” image stacks. Separately for each moving image stack, translation transformation was used to resolve linear shifts in *x*-, *y*-, and *z*-direction. Next, non-rigid three-dimensional b-spline registration was applied to determine local tissue deformations (Klein et al., 2007; Metz et al., 2011). Then the computed transformation parameters were applied to the cell stacks of 2 and 4 dpi, respectively. Finally, a 3D median filter was used (2-pixel radius in *x*, *y*, *z*) to cover technical noise. Eventually, the registered blood vessel stacks of all time points and the cell stacks in two RGB image stacks were merged to evaluate cell migration over time (see Supplementary Figures 4b,b’,c,c’).

### Hypertrophy Analysis

#### Volume Estimation and Statistical Analysis

Our single-cell volume estimation is an adaptation of our method described previously (Levine and Reynolds, 1999). For each image stack, slices were smoothed with a two-dimensional Gaussian filter (sigma = 0.5) to remove noise. Cell somata were identified by three-dimensional thresholding using the ImageJ plugin 3d object counter v2.0 (Bolte and Cordelieres, 2006). Thresholds were automatically determined using Otsu’s method (Otsu, 1975). When necessary, the thresholds were adjusted so that only the cell soma and the main processes were identified as the foreground object. Volume ratios for all cells were calculated by dividing the volume of the later time point (2 dpi) by the respective volume at day 0. The three different groups (hypertrophic, non-hypertrophic, and control) were statistically tested using one-way ANOVA combined with a Tukey post-test, as the cells showed Gaussian distribution. Statistics was performed with GraphPad Prism 5.0.

#### Gaussian Mixture Model Comparison

We assessed the number of Gaussian distributions necessary to fit the volume fold change of *n* = 116 single cells after injury with a variational Bayes expectation maximization approach using the pmtk3 toolbox (Murphy, 2012); 64 cells considered hypertrophic and 52 cells showing no hypertrophy from 10 animals were analyzed (Supplementary Figure 1c) as well as 28 control cells from three animals (Supplementary Figure 1d). Summarizing, we compare the likelihoods of mixture models with up to six different populations, optimize the respective parameters of the Gaussian distributions, and find k = 2 populations as a best fit for the observed data (see Supplementary Figures 1c,d).

### Immunohistochemistry

**FIGURE 5:**
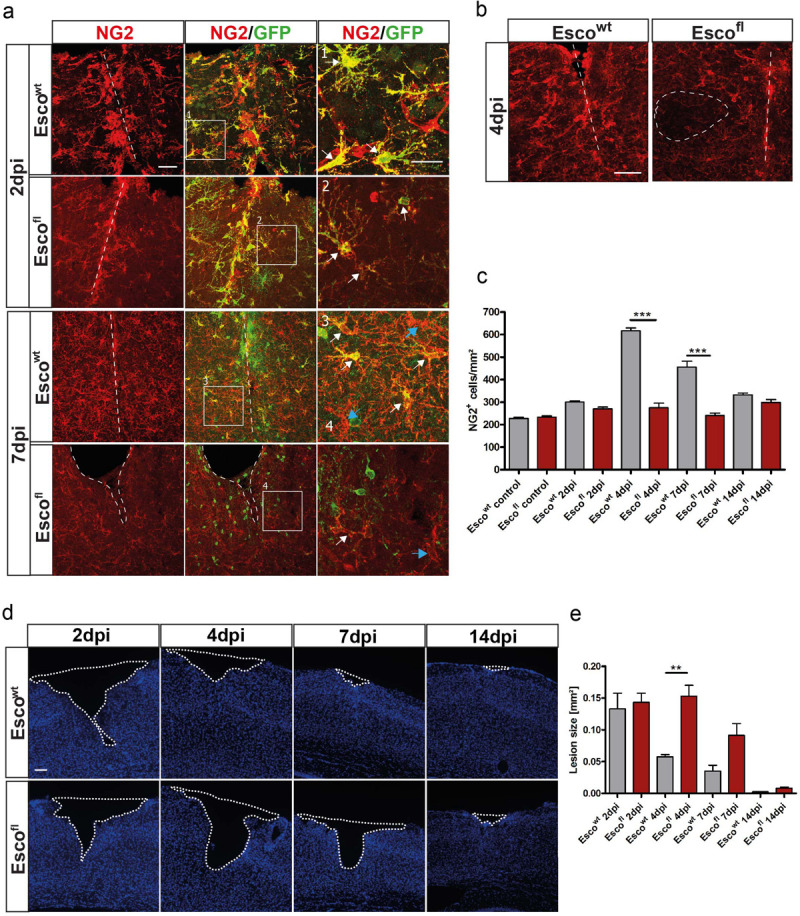
Depletion of NG2-glia after injury leads to impaired wound closure. **(a)** Confocal images of NG2^+^ cells in Esco^wt^ and Esco^fl^ animals at 2 and 7 dpi. **(b)** NG2^+^ cells in Esco^wt^ and Esco^fl^ animals at 4dpi. In Esco^fl^ animals, areas with complete absence of NG2-glia can be observed (dashed ellipse). **(c)** Cell counts of NG2^+^ cells per mm^2^ in Esco^wt^ and Esco^fl^ animals in control, non-lesioned brains and at different timepoints after the lesion. Esco^fl^ mice show a reduced cell number after injury (n = 3 animals for each genotype and timepoint, cell counts are presented as mean + SEM; 1way ANOVA with Tukey post-test: ^∗∗∗^ indicates significance level of *p* < 0.0001). **(d)** Lesion size in the cerebral cortex visualized by the lack of DAPI positive cells in Esco^wt^ and Esco^fl^ animals at different timepoints after the lesion. **(e)** Size of the lesion in mm^2^ at 2, 4, 7 and 14dpi in Esco^wt^ and Esco^fl^ animals. Esco^fl^ animals show a significantly bigger lesion compared to Esco^wt^ control littermates. (n = 3 for Esco^wt^ (2, 7 and 14dpi), n = 4 for Esco^wt^ (4 and 14dpi), n = 5 for Esco^fl^ (2dpi), n = 6 for Esco^fl^ (4 and 7dpi) animals), data are presented as mean±SEM; 1way ANOVA with Tukey post-test: ^∗∗^ indicates significance level of *p* < 0.001. Scale bars represent 25μm in **(a)** inlays, 50μm in **(a)** and **(b)**, 100μm.

Animals at different time points after the injury (2, 4, 7, and 14 dpi) were anesthetized and transcardially perfused with 4% paraformaldehyde (PFA). The collected brains were postfixed in 4% PFA for 30 min followed by cryoprotection in 30% sucrose; 30-μm-thick sections were cut and stained as previously described (Busch et al., 2010; Sofroniew and Vinters, 2010) with the following primary antibodies: rabbit (rb)-NG2 (1:500, AB5320 Millipore), mouse (m)-GFAP (1:500, G3893 Sigma–Aldrich), and chick-GFP (1:500, GFP-1020 Aves Lab). According to the primary antibodies, fluorochrome conjugated secondary antibodies were chosen: anti-rb Cy3 or A647 (1:500, 711-165-152 or 111-605-144 Dianova), anti-m Cy3 or Dylight 649 (1:500, 115-165-003 or 115-496-072 Dianova), and anti-chick A488 (1:500, A11039 Life Technologies). Additionally, nuclei were stained with DAPI (4’,6-diamidino-2-phenylindole, 1:10,000, D9564 Sigma–Aldrich). Multi-channel confocal images were obtained using a Zeiss confocal microscope system (LSM 710) and analyzed using the cell counter plug-in for FIJI^1^ (based on ImageJ 1.48i). The cell number analysis (Figure 5) was performed on three sections of three animals for each time point. The area spanning 50 μm surrounding the lesion core (identified using GFAP staining) was counted until up to ∼350 μm below the pial surface with an image depth of ∼10 μm. For Figure 5c, a total number of 1828 cells were counted. For the lesion size, ≥ 5 sections of ≥ 3 animals were analyzed. To measure the size, pictures of the DAPI channel were acquired and the DAPI-free area in the cerebral cortex was measured using the Fiji software. For the direct comparison, only the biggest lesion of each animal was considered.

### Statistics

A statistical analysis was performed on the non-pooled datasets. Results are represented as means or as mean ± SEM. The sample size (*n* ≥ 3 animals) was justified by experience from previous studies and no exclusion of data points or datasets were performed. For the analysis, no randomization was performed and the investigator was not blinded to the group allocation during the experiment or analysis. We expect our data to be normally distributed and, used an unpaired *t*-test or one-way ANOVA with Tukey post-test for grouped analysis. For the data which were not normally distributed, Wilcoxon rank sum test was used. Data were considered as significant with *p* < 0.05^∗^, *p* < 0.01^∗∗^, and *p* < 0.0001^∗∗∗^. A statistical analysis was performed with GraphPad Prism 5.0.

## Results

### NG2-Glia Reaction Upon Injury

To study the behavior and reaction of single NG2-glia after injury, we performed *in vivo* time-lapse two-photon imaging of injured Sox10iCreER^*T*2^ x CAG-eGFP mice, which express the GFP protein in the oligodendrocyte lineage after tamoxifen administration (Figure 1a). After induction of adult mice, we performed a small, punctate wound injury (PWI) (Bardehle et al., 2013) in the somatosensory cortex with a straight ophthalmic knife. Subsequently, a cranial window was implanted, and, each time before imaging, the blood vessels were labeled by intravenous injection of Texas Red^TM^ conjugated Dextran, in order to establish landmarks to follow cells of interest over time (Figure 1). The first imaging session was performed 45 min after injury (0 dpi; Figure 1a and Supplementary Movie 1). We then repetitively imaged these cells at different time points after injury (2–28 dpi; Figures 2a–c) and analyzed their behavior. At 0 dpi, most NG2-glia showed their typical ramified morphology with radially distributed processes (Figure 1b). Most of NG2-glia reacted to the injury, starting to fill the lesion core already at 2 dpi, overcoming the known homeostatic control of NG2-glia (Hughes et al., 2013) (Figures 1b,c,e). In contrast to previous reports, we observed almost no cell death of NG2-glia after injury as we could follow almost all cells within the first time points (Figures 1b–d). Additionally, mature oligodendrocytes did not show any observable response to the injury (arrows in Figures 1b–d and Supplementary Movies 4, 5). To further characterize the behavior of NG2-glia, we defined and classified their reaction types in the following categories: (a) hypertrophy, representing the enlargement of the cell body, main processes volume, or both (Figure 1b′ and Supplementary Figure 1), (b) polarization determined as the reorganization of their morphology into an elongated cell (process/es, soma, or both) toward a specific direction (Figure 1c′), (c) migration, defined as the movement of the cell body for at least 10 μm between two time points (Figure 1c, Supplementary Figure 5, and Supplementary Movie 2), (d) proliferation (Figures 1d′,e,f and Supplementary Movie 3), and (e) combined when at least two of the aforementioned responses occur (Figures 1d,e′,f).

When we analyzed all cells up to 500 μm away from the lesion, we observed a fast and heterogeneous reaction in the majority of NG2-glia (188 of 254 cells in eight mice; Figure 1e) already at 2 dpi, showing one or more of these reaction categories and they continue to respond in a similar degree at 4 dpi (Figure 1f). However, the number of migratory, proliferating, and static cells increased as time advanced. In contrast, the number of hypertrophic, polarizing, and combined reaction cells decreased (compare Figure 1e with Figure 1f).

### NG2-Glia Undergo Morphological Changes After Injury

Our analysis revealed the peak of NG2-glia reactivity within the first 4 dpi, which gradually dropped between 1 and 4 weeks after injury (Figure 2).

For example, hypertrophy was rather a quick and transient event, peaking at 2 dpi where 42% of the NG2-glia were hypertrophic (106 out of 254 cells in eight mice), declining to 27% at 4 dpi (63 out of 222 cells, in six mice), and almost disappearing at 6 dpi (4 out of 114 cells; Figure 2d). Interestingly, 75% of the traceable cells with hypertrophic morphology at 2 dpi kept their altered morphology until 4 dpi (47 out of 63 cells; red bar, Figure 2d), while only around 7% of NG2-glia developed hypertrophy for the first time between 2 and 4 dpi (16 out of 222 cells; green bar; Figure 2d). Volumetric analysis of hypertrophic NG2-glia revealed that these cells displayed a threefold size increment on average compared to non-hypertrophic ones within the proximity of the lesion or cells from non-injured brains (64 hypertrophic vs. 52 non-hypertrophic cells, in 10 injured animals and 28 cells from three non-injured animals; Supplementary Figures 1a,b). Interestingly, the frequency distribution confirmed the existence of hypertrophic and physiological non-hypertrophic populations showing a high overlap between visual and statistical classification (Supplementary Figures 1c,d). Notably, hypertrophic NG2-glia at 2 dpi tended to proliferate at 4 dpi (42 ± 6%; Supplementary Figure 2), while almost half of the cells reduced their size at this time point (47 ± 7%, Supplementary Figure 2).

Like hypertrophy, polarization reached its peak reaction already at 2 dpi, albeit decreasing only after 11 dpi (Figure 2e). Interestingly, polarization seemingly affects a specific population of NG2-glia because the maximum recruitment of cells occurs immediately at 2 dpi, adding only a few new cells to the response later (Figure 2e). When we analyzed the direction of the cell processes, we observed that while in the first 4 dpi most NG2-glia polarized toward the injury site, later, they changed their orientation, shifting to a more random (away from the injury site) direction (Figure 2f). As expected, polarized NG2-glia at 2 dpi tended to migrate (40 ± 7%) at 4 dpi, whereas radially symmetric cells did not (10 ± 4%; Supplementary Figure 3). However, more than half (60 ± 7%) of the polarized cells did not migrate at all, but instead lost their polarization at 4 dpi (41 ± 8%; Supplementary Figure 3), suggesting that polarization does not necessarily lead to migration. We could not observe a clear event connection between polarization and hypertrophy or proliferation (Supplementary Figure 3). These results highlight a fast and transient morphological reaction of NG2-glia shortly after injury, followed by a steady return to physiological levels already after a week.

### Migration and Proliferation of NG2-Glia After Injury

Similar to the morphological changes of NG2-glia, migration toward the injury site started shortly after acute lesion (Figures 1c′, 2h). To assess whether the observed migration was an active process and not just cells displaced due to tissue contraction, we superimposed images of the same cells at different time points after registration of the stacks, corroborating active migration of NG2-glia (Supplementary Figure 4 and Supplementary Movies 4–7).

In contrast to the relatively fast and transient effects on the morphology, migratory behavior lasted longer. Notably, the maximum speed and distance of migrating NG2-glia remained at their highest between 2 and 11 dpi, declining only thereafter (Figure 2h and Supplementary Figures 5e,f). As the imaging intervals became longer after 11 dpi, relatively slow-moving cells were also considered as migrating cells as long as they kept their directionality. Although the overall number of migrating cells did not drastically decline at later time points, NG2-glia changed the migration direction, and their maximum migration distance as well as their velocity returned to control levels (Supplementary Figures 5e,f). In contrast to the polarization, we observed that not only previous migrating cells kept on moving (“old,” red bars, Figure 2h and Supplementary Figure 5d) but also new cells started to migrate at later time points (“new,” green bars; Figure 2h). These data suggest that migratory behavior is the most prolonged response of NG2-glia after injury.

Interestingly, migrating cells at 2 dpi showed a stronger reactivity at 4 dpi than the stationary NG2-glia, with a higher proportion of cells becoming hypertrophic or polarized (51 ± 4 and 53 ± 8%, respectively; Supplementary Figure 5d). The direction of migrating NG2-glia correlated with the orientation of the polarized processes that preceded the change in migration direction. Notably, most migrating cells no longer moved toward the injury site at 8 dpi (Figures 2f,i). These data further corroborate that NG2-glia indeed exhibit directional migration toward the injury site within the first week after the lesion, temporally filling the wound before the movement direction returns to a more randomized orientation within the brain parenchyma.

Proliferation increased at 2 dpi and peaked at 4 dpi, and was the latest feature to peak when compared to the other cellular responses (Figure 2). Thereafter, the percentage of dividing NG2-glia abruptly declined and already reached baseline levels between 8 and 11 dpi (Figure 2g). Distal to the injury, we did not observe two proliferation events in a same cell between two time intervals (0 out of 72 dividing cells at all time points analyzed; Supplementary Figure 6d). Nevertheless, in a very small number of cases adjacent or within the injury core, some cells had divided multiple times between 2 and 4 dpi, resulting in three daughter cells (2 out of 72 proliferating cells from six animals). However, the massive increase of NG2-glia within and juxtaposed to the injury core between two time points (Figures 4a,b) suggests that cells most likely undergo more than one round of cell division, which cannot be adequately assessed due to the high cellular density and reactivity in these areas in comparison to regions distal to the injury. After cell division, typically both daughter cells polarized in opposite directions, migrating apart from each other (Supplementary Figures 6a,b). However, we also found cases in which both cells showed a polarization toward the same area (in PWI direction 5 out of 72 dividing cells; Supplementary Figure 6c). The degree of polarization and migration varied, with some progeny showing weak or almost no polarization or staying nearby over time (Supplementary Figure 6). Notably, a considerable proportion of NG2-glia showed hypertrophy before proliferation, suggesting cells might need to increase in volume before dividing. Here we could show that the increase in NG2-glia number after injury results from directional migration and enhanced proliferation of these cells.

### Injury Size and Distance Influence the Degree of NG2-Glia Reaction

About one-quarter of the imaged NG2-glia did not react in any detectable manner upon PWI (Figures 1e,f). It is possible that NG2-glia reaction depends on peripheral molecular signals or infiltrating immune cells from the bloodstream, which diffuse into the brain parenchyma after the lesion has compromised the integrity of the BBB. Accordingly, we expected that a bigger injury would increase the probability of distal NG2-glia to be exposed to such factors. To test this hypothesis, we performed a larger SWI (dimensions: ∼1 mm in length; Bardehle et al., 2013; Figures 3b, 4d–f) and compared the NG2-glia response here to the smaller PWI (∼100 μm in length; Figures 3a–c). In the SWI, we could observe a dramatic decrease in the proportion of static cells at 2 dpi (10 ± 2% compared to 26 ± 5% after PWI; compare Figures 1e and 3d) and an increase in the number of NG2-glia migrating and proliferating (Figure 3c).

If diffusible signals are released from the lesion area, we would assume that the cell responsiveness should differ according to their proximity to the injury. Therefore, at 2 dpi, we analyzed the NG2-glia reaction in relation to their distance to the PWI, and we found a negative correlation between distance to the site of injury and response of NG2-glia with cells further away from the wound showing a weaker reaction (Figures 3e–g). Furthermore, cells reacted stronger within a distance of 200 μm around the injury, at 2 dpi (Figure 3f). At 4 dpi, this correlation decreased with the proportion of polarizing and migrating cells within 50–100 μm remaining higher (Figure 3g). In contrast to the other responses, the percentage of proliferating cells did not depend on the distance to the injury, neither at 2 nor at 4 dpi. These results suggest that the recruitment of NG2-glia as a reaction following an injury only takes place if the cues released from the injury site reach a certain distance, albeit, proliferation could be an exception to this rule.

### NG2-Glia Fill the Injury Core by Migration, Proliferation, and Hypertrophy

As described above, the proximity to the injury influences NG2-glia reaction; hence, we expected that cells located in the lesion core would show the most robust response. Already at 2 dpi, NG2-glia reaction was strong after PWI (Figures 4a,a’ and Supplementary Movie 8) for those cells that were traceable over time. Here it is worth noting the substantial differences between NG2-glia that are located in the area surrounding and within the core of the injury (compare Figures 2d–i with 4b). First, from the 18 identifiable cells (seven animals) within the injury core, all NG2-glia reacted within 2 dpi (Figures 4b,c) with hypertrophy, migration, proliferation, or combined responses, while only a minority showed an apparent polarizing reaction (29% at 2 dpi and no cells at 4 dpi; Figure 4b). Second, contrary to NG2-glia located distal to the injury, the highest degree in proliferation was shown after 2 dpi and then is reduced again after 4 dpi (compare Figure 2g with Figure 4b). Third, hypertrophy increases and remains elevated until 4 dpi, and it is not reduced like in cells distal to the injury (compare Figure 2d with Figure 4b). While all cells in the injury core reacted within 2 dpi in the PWI (Figure 4c), in the larger SWI, where also the lesion area which had to be covered is much bigger, NG2-glia needed a longer time to fill up this area (4 days; Figures 4d–f). In both lesion paradigms, these cells began to slowly diminish in number at later time points. By 28 days, the lesion core area resembled an uninjured area in terms of NG2-glia morphology and distribution (Figure 2c), as also confirmed by immunohistochemistry of still images (data not shown).

### Depletion of NG2-Glia Leads to Delayed Wound Closure

As we observed this substantial accumulation of NG2-glia, a product of cells proliferating and migrating into the injury core as a very robust and reproducible response, we wanted to assess the function of NG2-glia after injury. Therefore, we specifically ablated proliferating NG2-glia after injury by taking advantage of the Esco2^*fl/fl*,^ (Whelan et al., 2012) and the Sox10iCreER^*T*2^ x CAG-eGFP mouse lines, which induce cell death of recombined cells during cell division, and thus, lead to a depletion of proliferating NG2-glia (Schneider et al., 2016; Hesp et al., 2018). This depletion is especially prominent after injury, when many NG2-glia are proliferating, as it decreases recombined NG2-glia (Figures 5a–c) and thus diminishes the total NG2-glia population (Figures 5a–c). Even though non-recombined NG2-glia can partly counteract this effect by compensatory proliferation, the overall number of NG2-glia around the injury is abrogated in Esco2^*fl/fl*^ mice (Figures 5a–c). We hypothesized that this rapid and transient increase in NG2-glia cell number plays a role in wound closure. Indeed, although the lesion size was similar between all genotypes at 2 dpi, the observed reduction in the number of NG2-glia in the Esco2^*fl/fl*^ mice led to impaired wound closure, showing more significant lesion areas at 4 and 7 dpi compared to control littermates (Figures 5d,e). Interestingly, these differences in lesion size were restored at 14 dpi when NG2-glia cell numbers were almost completely recovered (Figures 5c,e). Notably, a change in BBB permeability could not be detected (data not shown). Thus, a reduction of NG2-glia within and around the injury core led to an impairment and delay in wound closure, implicating an essential role of NG2-glia for tissue repair after injury.

## Discussion

NG2-glia in the adult brain do not just proliferate in the cerebral cortex parenchyma but can also differentiate into oligodendrocytes (Dimou et al., 2008; Kang et al., 2010; Vigano et al., 2013; Young et al., 2013). After an acute or chronic injury in the adult, CNS NG2-glia become reactive (Levine and Reynolds, 1999; Hampton et al., 2004), changing their morphology and upregulating the proteoglycan NG2 strongly (Levine, 1994), as well as increasing their proliferation rate (Keirstead et al., 1998; Buffo et al., 2005; Zawadzka et al., 2010; Behrendt et al., 2013). Although the reaction of NG2-glia after an injury is well characterized by the analysis of still images from post-mortem samples, many questions regarding the dynamic behavior of these cells remain open. Therefore, we performed a time series with *in vivo* two-photon laser scanning microscopy following TBI to study the temporal response of NG2-glia. We found that NG2-glia react rapidly (within 2 dpi) and heterogeneously by hypertrophy, polarization, and migration toward the injury, while proliferation is a later event, occurring mainly between 2 and 6 dpi. Although their reaction involves a broad spectrum of responses and depends on the distance to the injury, most NG2-glia showed at least one of these reactions. Notably, blocking the increase in NG2-glia numbers by specifically ablating proliferating cells led to wound closure deficits, highlighting the importance of their role after injury.

**FIGURE 6:**
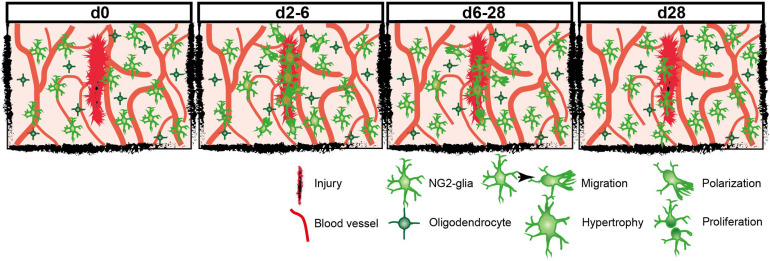
Model scheme of the reaction of NG2-glia at different timepoints after injury.

Under physiological conditions, NG2-glia are evenly distributed, building a network throughout the brain by maintaining their exclusive domains through self-repulsion, which prevents long distance migration of the cells (Hughes et al., 2013). Although they can differentiate and undergo apoptosis, NG2-glia maintain a constant density by proliferation and short-range migration (Hughes et al., 2013). However, after injury, the enhanced proliferation and migration of NG2-glia toward the lesion lead to a higher cell density, arguing that cells transiently overcome their homeostatic distribution (Hughes et al., 2013). Within and adjacent to the core of the injury, cells were extremely responsive, increasing in number (Figure 4) without many apoptotic events. This contrasts largely with previous observations in laser-induced injury, which observe that the total population remains unchanged by coupled apoptosis and cell-renewal (Hughes et al., 2013). Quite the contrary, we observed here that the physiological cell density is only restored 4 weeks after injury by reorientation of NG2-glia away from the wound, starting 1 week after the traumatic insult and showing a progressive normalization of cell numbers overtime (Figures 2, 6). Thus, we are tempted to suggest that the homeostatic distribution of NG2-glia is restored only later after the primary damage from TBI took place and not during the insult.

The observed change in proliferation does not appear to be restricted to TBI but has also been shown in AD mouse models (Behrendt et al., 2013) and multiple sclerosis (MS) in human patients (Maeda et al., 2001; Cui et al., 2013). However, even though NG2-glia density increases after TBI and AD, the number of progenitors is strongly reduced within chronic demyelinating lesions in MS (Chang et al., 2002; Sim et al., 2002). These differences might reflect a failure of NG2-glia to sense the environment and react to the changes in myelin levels by differentiating into oligodendrocytes or to respond to a growing environmental hostility inhibiting the reaction of these cells. The differences observed in the impairment of NG2-glia homeostasis justify a future search for the signals influencing or maintaining this homeostasis.

During development, NG2-glia also show directed migration along axonal tracts over long distances before differentiating into oligodendrocytes (Kessaris et al., 2006). Displacement of NG2-glia involves continual remodeling of their cytoskeleton that can be controlled by Rho-GTPases, like cdc42, RhoA, and Rac (Etienne-Manneville and Hall, 2002). While the deletion of cdc42 did not affect NG2-glia migration or proliferation *in vitro* (Thurnherr et al., 2006), a study could show that the proteoglycan NG2 itself can control migration by regulating planar cell polarity via RhoA/ROCK pathway activation (Biname et al., 2013). Indeed, the same research observed in still images that NG2-glia in NG2-knockout mice that have sustained an SWI, no longer polarize to the same extent toward the injury. Interestingly, NG2-knockout animals have exacerbated astrocyte reaction, prolonged BBB disruption, and slower resolution of the lesion after undergoing an SWI (Huang et al., 2016), similar to the impairment in wound closure observed in mice with ablated proliferating NG2-glia (Figure 5).

Ischemia patients have shown to have increased levels of extracellular glutamate that can be prolonged for several days (Bullock et al., 1995), a neurotransmitter that has been shown to regulate NG2-glia migration (Gudz et al., 2006). Other mechanisms may include bFGF acting as a chemo-attractant that could be released, e.g., by reactive astrocytes following different pathological insults such as demyelination in MS or cortical injury (Rowntree and Kolb, 1997; Clemente et al., 2011). Interestingly, immunohistochemical studies revealed a gradient of bFGF with high cytokine levels in the core of the injury and low levels more laterally (Biname et al., 2013). VEGF, released by endothelial cells after injury, is another candidate for promoting the migration of NG2-glia (Hayakawa et al., 2011). Thus, our observations that NG2-glia do exhibit directional migration toward the injury site, in contrast to astrocytes (Bardehle et al., 2013), now lay the basis for the search of molecules mediating this response.

While NG2-glia in the healthy adult gray matter divide slowly with a cell cycle length of several weeks (Psachoulia et al., 2009; Simon et al., 2011; Clarke et al., 2012), NG2-glia upon traumatic injury re-enter the cell cycle rapidly and show a substantial increase in proliferation (McTigue et al., 2001; Buffo et al., 2005; Simon et al., 2011). In this work, we showed that most NG2-glia only divide once, and, together with their migration, result in cell accumulation at the wound core. However, the limited resolution in the core restricts the precise assessment on this area. Notably, in contrast to all other observed responses, proliferation of NG2-glia appeared relatively late (peak at 4 dpi), and its magnitude was neither dependent on the injury size nor the distance to the injury. However, within the injury core, proliferation is faster and more robust than in the NG2-glia outside the wound. This evidence shows that factors that regulate NG2-glia proliferation probably do not diffuse into the brain parenchyma or that they respond solely to the physical impact of the object that produces the TBI. The signals mediating NG2-glia proliferation after injury are still unknown and have to be further analyzed. Although the migratory response exceeds proliferation in scale and duration, the ablation of proliferative NG2-glia has a massive effect on wound closure (Figure 5), suggesting the importance of an increase in the number of NG2-glia. This increase in proliferation can also be observed in other types of injury, e.g., chronic plaque deposition (in general models of AD) or demyelination (Keirstead et al., 1998; Behrendt et al., 2013). However, as also shown for astrocytes and in contrast to microglia, which react to all kinds of brain injuries, NG2-glia proliferation seems to be often triggered by lesions that show a disruption of the BBB, while the ablation of half of the neurons in the adult mouse cerebral cortex (Radde et al., 2006) does not lead to a change of NG2-glia proliferation (Sirko et al., 2013). This observation, therefore, strongly supports the idea that factors in the blood could influence the reaction of both macroglial cell types reacting to injuries: NG2-glia and astrocytes.

In contrast to very homogenous behavior under physiological conditions (Hughes et al., 2013), NG2-glia in the somatosensory cortex have a broad spectrum of different behaviors after TBI. It is still unclear to which extent these distinct behaviors are due to subsets of NG2-glia that are intrinsically different or due to the specific local environment influencing their particular behavior. However, as reacting cells show a heterogeneous behavior even within the same area around the lesion where they should receive comparable input of released factors and signaling molecules, the environmental influence alone is unlikely. In contrast, intrinsic heterogeneity has already been observed between NG2-glia in the gray and white matter of the cerebral cortex (Vigano et al., 2013). The extent to which NG2-glia are also heterogeneous within the same region, like here in the cerebral cortical gray matter, is still unknown. However, expression of the G-protein coupled receptor, GPR17, could only be observed in a subset of NG2-glia in the adult cerebral cortex (Boda et al., 2011). Interestingly, an injury increased the differentiation rates of this GPR17 positive subset (Vigano et al., 2016). Another explanation of these distinct reactions of NG2-glia may be that these are in different phases of their cell cycle or maturation state. Most likely, the response of NG2-glia results from a combination of intrinsic heterogeneity and local differences in the environment leading to these strikingly different responses.

While the majority of NG2-glia around the injury core showed a large variety of reactions, some NG2-glia did not show any observable change (static cells; Figures 1e,f, 3d). Likewise, mature oligodendrocytes labeled by our mouse model were remarkably stable over time. Even in close proximity of the injury, oligodendrocytes never showed any drastic morphological changes and disappeared on only a few occasions. Our data showed that oligodendrocytes probably do not contribute to scar formation or wound healing.

On the contrary, the fast-reacting NG2-glia might contribute to the first cellular scaffold built early after injury. However, the question remains what is the exact role of the NG2-glial reaction? Our observation that the ablation of proliferating NG2-glia, and hence, the lack of increase in NG2-glia numbers within and around the lesion site impairs and delays wound closure (Figure 5) suggests that they indeed participate in the first steps of tissue remodeling and healing following acute injury in the brain. This function is supported by experiments in spinal cord injury that NG2-glia ablation, prior to a hemisection lesion shows delay in wound closure (Hesp et al., 2018). Nonetheless, in this work, we have not depleted the whole population but only the proliferating population, preventing the increase of NG2-glia numbers at the lesion site, perhaps reflecting different functions in tissue healing. Furthermore, it is also possible that these proliferating cells might be a specialized subset of NG2-glia that secrete or even specifically respond to factors that promote regeneration and wound closure at the lesion site.

It is likely that further roles and cell–cell interactions take place in addition to the observable physical scaffold formation. These functions are presumably mainly secondary functions occurring at later stages that might be necessary for wound healing and tissue remodeling. In general, these complex and multifaceted events in tissue regeneration after an injury have stereotypic components, such as systemic response and extracellular matrix deposition, which are shared between various tissue types. The apparent privileged nature of the CNS with its distinct cellular composition results in many tissue-specific events, and an insufficient regenerative capacity (Shechter and Schwartz, 2013). This hosts detrimental consequences for the majority of CNS pathologies. Thus, it is essential to maximize our understanding of the cellular components and the underlying mechanisms contributing to the regenerative response in the brain.

In summary, NG2-glia reaction increased depending on the injury size and distance from the lesion core, whereas some cellular responses like polarization showed a stronger correlation than proliferation. Overall, NG2-glia respond to injury with a fast and robust reaction that mainly takes place within the first 2–6 dpi leading to a significant increase in cellular density directly within as well as in close vicinity to the injury core. Neighboring NG2-glia replace migrated cells by increased proliferation. One week after injury, the reactivity has already decreased, and cellular density and distribution started to return to physiological levels. Between 3 and 4 weeks after TBI, the morphology and distribution of NG2-glia fully returned to physiological conditions (Figures 2, 6). This work strengthens the role of NG2-glia in wound closure and signaling to other cell types that respond at later time points, such as astrocytes.

## Data Availability Statement

The original contributions presented in the study are included in the article/Supplementary Material. Further inquiries can be directed to the corresponding author.

## Ethics Statement

The animal study was reviewed and approved by Regierung Oberbayern, Munich, Germany.

## Author Contributions

AS performed the experiments and data analysis, designed the experiments, and wrote the manuscript. SJ performed the experiments with Esco2^*fl*^ mice. CS initially designed and performed the experiments and analyzed the results. FB and CM assisted in data processing and volume analysis (Supplementary Figures 1, 2). JEvB discussed the results and wrote the manuscript. LD designed and supervised the project and experiments, discussed the results, wrote the manuscript, and coordinated and directed the project. All authors contributed to the article and approved the submitted version.

## Conflict of Interest

The authors declare that the research was conducted in the absence of any commercial or financial relationships that could be construed as a potential conflict of interest.

## Footnotes

1 http://fiji.sc/Fiji
