# Supplementary material for: NG2-Glia Transiently Overcome Their Homeostatic Network and Contribute to Wound Closure After Brain Injury
Source: Front Cell Dev Biol. 2021 Apr 27;9:662056. doi: 10.3389/fcell.2021.662056 (PMC8128074; doi:10.3389/fcell.2021.662056)
Supplement: Supplementary file 1 [file Data_Sheet_1.PDF]

Supplementary Information

NG2-glia transiently overcome their homeostatic network and contribute to wound closure after brain injury

Axel von Streitberg, Sarah Jäkel, Jaime Eugenin von Bernhardi, Christoph Straube, Felix Buggenthin, Carsten Marr and Leda Dimou

Supplementary Figure 1. Cells classified as hypertrophic show a significant difference in volume fold change. (a) Exemplary images for a hypertrophic (top row) and a non-hypertrophic (bottom row) NG2-glia at two succeeding timepoints. (b) Boxplot comparison of n=64 hypertrophic and n=52 non-hypertrophic NG2-Glia from 10 different mice with injury and n=28 cells from 3 control animals without a lesion (Values represented as mean with whiskers extended to maximum and minimum of data). Cells classified as hypertrophic show a significant difference in fold change in comparison to non-hypertrophic and control cells. In contrast, the difference in the volume fold change of non-hypertrophic versus control cells is not significant (Wilcoxon Rank Sum Test: hypertrophic vs. control: $p=8{,}2653e^{-11}$; hypertrophic vs. non-hypertrophic: $p=2.7023e^{-13}$; non-hypertrophic vs. control: $p=0.8520$). (c) A Gaussian mixture model with two populations best describes the volume fold change of 116 cells after injury. The average fold change μ of the non-hypertrophic population (solid red fit) is 0.98, the average fold change μ of the hypertrophic population is 3.31 (dashed red fit). We determined the fold change threshold between the two populations as the intersection of the two distributions at 1.70. (d) The average fold change μ in a control set (n=28 cells from a non-injured sample) is 0.97. The 95 percentile of a fitted Gaussian distribution to the control population is 1.57 and can also be used as a fold change threshold. The two statistically determined fold

change thresholds lead to a hypertrophic and a non-hypertrophic subpopulation that overlap with 88% (Gaussian mixture model) and 94% (95 percentile of the control set) with the visual classification, respectively. Scale bars represent 10µm.

Supplementary Figure 2. Examples of hypertrophic NG2-glia and their further behavior. (a) NG2-glia (white arrow) next to a vessel, moving away from the vessel and showing a hypertrophic morphology at 2dpi migrates further and loses its hypertrophic morphology at 4dpi. (b) Hypertrophy at 2dpi can also be followed by cell division. (c) A cell (white arrow) getting hypertrophic at 2dpi and remaining hypertrophic until 4dpi without any further detectable reaction. (d) Proportion of cells that were hypertrophic (red) or not (blue) at 2dpi and their further reaction at 4dpi (n=6 animals; data are presented as mean). Images show maximum intensity projections of 30 (a: d0 and d2, c) or 40 (a: d4, b) µm deep stacks. Scale bars represent 20µm.

Supplementary Figure 3. Examples of polarizing NG2-glia at 2dpi and their reaction at 4dpi. NG2-glia showing polarization at 2dpi can retract their processes and not show any further reaction (a,b) or changes its polarization (c) at 4dpi. (d) Proportion of cells that showed polarization toward the injury (red) or no polarization (blue) at 2dpi and their further reaction at 4dpi (White arrows indicating NG2-glia; yellow arrows indicating oligodendrocytes; n=6 animals; data are presented as mean). Images show maximum intensity projections of 30 (a,c) or 40 (b) µm deep stacks. Scale bars represent 20µm.

Supplementary Figure 4. Automated Registration of 3D image stacks of 0, 2 and 4dpi indicates migration of NG2-glia toward lesion site. (a) Pipeline for registration of image stacks using blood vessels as landmarks (for details, see the methods section): At every timepoint, the image stack is split into two grayscale stacks to separate the landmarks (blood vessels) from the data of interest (GFP^+ cells). (b) Overlay of z-projections from 0dpi (red), 2dpi (green) and 4dpi (blue) showing stained blood vessels. Linear shifts due to slight changes of the imaging angle and non-linear shifts due to tissue swelling are observable. (c) Overlay of z-projections from 0dpi (red), 2dpi (green) and 4dpi (blue) showing GFP-labeled NG2-glia. Due to the systematic shifts between the timepoints, observation of migration might be spurious. (b') Overlay of z-projections of blood vessel stacks. After non-rigid registration, the blood vessels of all timepoints are adequately aligned. (c') Overlay of z-projections of GFP^+ cells after transforming the stacks from 2dpi and 4dpi in accordance to the computed registration parameters from the blood vessel stacks. After registration migration of cells toward the lesion site between the different timepoints is clearly observable (white arrows). Images show maximum intensity projections of 30µm. Scale bars represent 40µm.

Supplementary Figure 5. Examples of migrating NG2-glia and their further reaction. (a) NG2-glia (white arrow) migrating over a vessel and showing a hypertrophic morphology. (b) Cell migrating until d2 followed by a cell division at 4dpi. (c) NG2-glia that keeps migrating over time. (d) Proportion of cells that showed a migratory behavior (red) or not (blue) at 2dpi and their further reaction at 4dpi (n=6 animals; data are presented as mean). Images show maximum intensity projections of 30μm deep stacks. Scale bars

represent 20μm. (e) Mean velocity (n=3-8 animals per timepoint; mean+SEM; μm per day) of migrating cells. (f) Maximum migration distance (n=3-8 animals per timepoint) of migrating cells.

Supplementary Figure 6. Examples of proliferating NG2-glia and their further reaction. (a-c) NG2-glia dividing at 2dpi mostly remain close to each other at 4dpi and partially polarize to opposite directions. (d) Proportion of cells that proliferated (red) or not (blue) at 2dpi and their further reaction at 4dpi (n=6 animals; data are presented as mean). Images show maximum intensity projections of 30 (a,c) or 24 (b) μm deep stacks. Scale bars represent 20μm.

Movie legends

Supplementary Movie 1: Live imaging of GFP^+ cells from the oligodendrocyte lineage and Texas-red labeled blood vessels in the somatosensory cortex of a $Sox10iCreR^{T2}$ x CAG-eGFP mouse. Stack of area surrounding the PWI (cell-free, black area in the middle of the image) at 0dpi starting from the dura (blue second harmonic signal). 175 images with a step size of 2μm and a total depth of 350μm; size=635.9x635.9μm. Magnification=20x, zoom=1.

Supplementary Movie 2: Live imaging of a migrating cell from 4dpi until 11dpi. Maximal projections of the cell at 4, 6, 8 and 11dpi. Depth of 20μm; size=125x84μm. Magnification=20x, zoom=1.

Supplementary Movie 3: Live imaging of a proliferating cell from 4dpi until 28dpi. Maximal projections of the cell at 4, 6, 8, 11, 21 and 28dpi. Depth of 30µm; size=91x99µm. Magnification=20x, zoom =1.

Supplementary Movie 4: Live imaging of eGFP$^+$ cells from the oligodendrocyte lineage and Texas-red labeled blood vessels in the somatosensory cortex of a Sox10iCreR^{T2} x CAG-eGFP mouse. Area surrounding the lesion at 0, 2 and 4dpi. Projections of stacks with the depth of 22.5µm; size=330x300µm. Magnification=20x, zoom =1.

Supplementary Movie 5: Superimposed projections of the 3 different timepoints (0dpi: green, 2dpi: blue; 4dpi: magenta) including the red channel (blood vessels) of 0dpi showing migrating NG2-glia and static oligodendrocytes (white arrows) after registration. Stack depth is 60µm; size=370x370µm. Magnification=20x, zoom =1.

Supplementary Movie 6: Superimposed 3D image of the red channel including the blood vessels of different timepoints (0dpi: red, 2dpi: green; 4dpi: blue) after registration. Stack depth is 183µm; size=635.9x635.9µm. Magnification=20x, zoom= 1.

Supplementary Movie 7: Superimposed 3D image of the green channel (NG2-glia and oligodendrocytes) of different timepoints (0dpi: red, 2dpi: green; 4dpi: blue) after registration based on the red channel. Stack depth is 183µm; size=635.9x635.9µm. Magnification=20x, zoom =1.

Supplementary Movie 8: Projections of stacks surrounding the lesion site at 0 and 2 dpi showing NG2-glia filling the injury core. Stack depth is 45µm; size=480x485µm. Magnification=20x, zoom= 1.
