# Supplementary figures and images for: NG2-Glia Transiently Overcome Their Homeostatic Network and Contribute to Wound Closure After Brain Injury
Source: Front Cell Dev Biol. 2021 Apr 27;9:662056. doi: 10.3389/fcell.2021.662056 (PMC8128074; doi:10.3389/fcell.2021.662056)

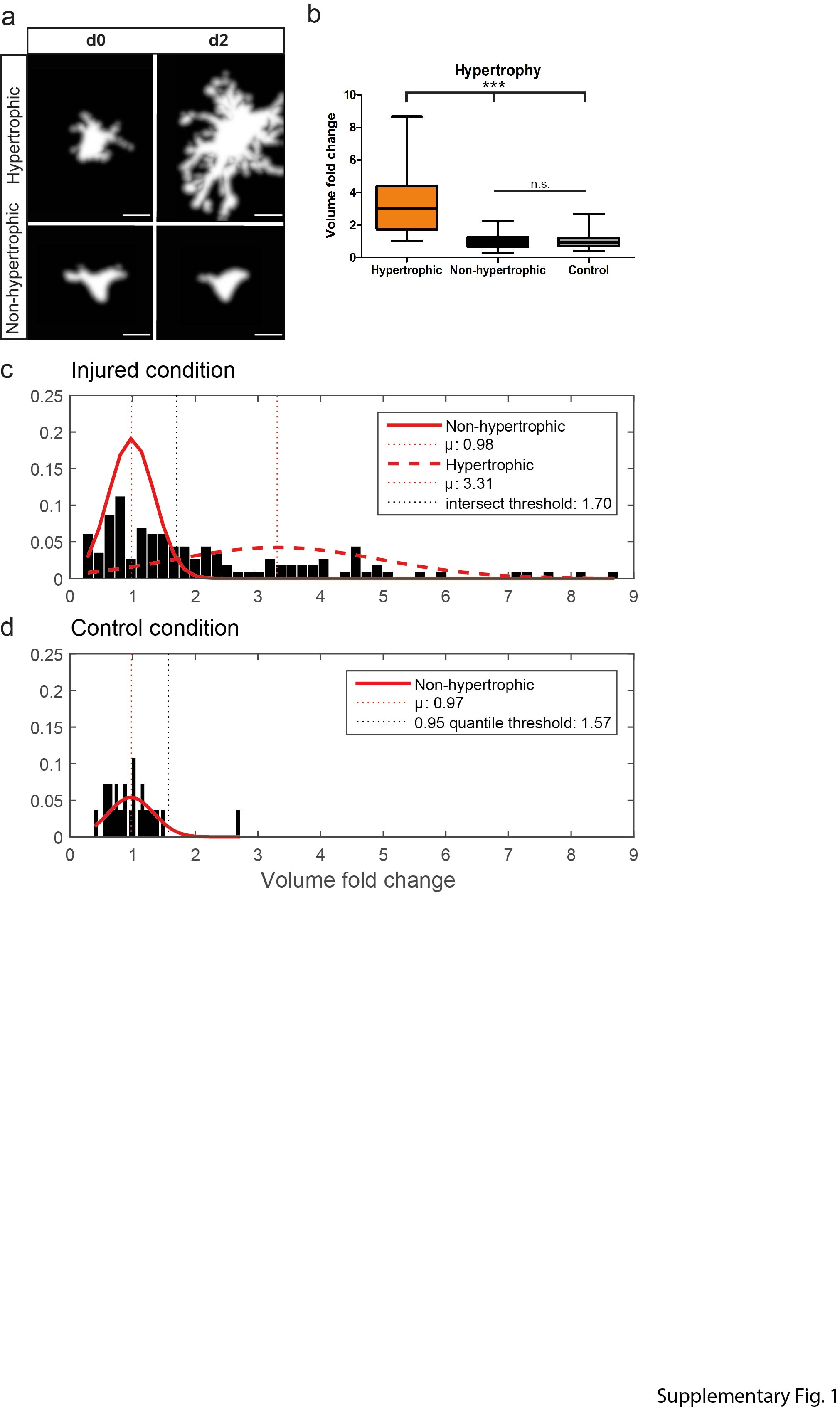

Supplement: Supplementary file 2 [file Image_1.JPEG]

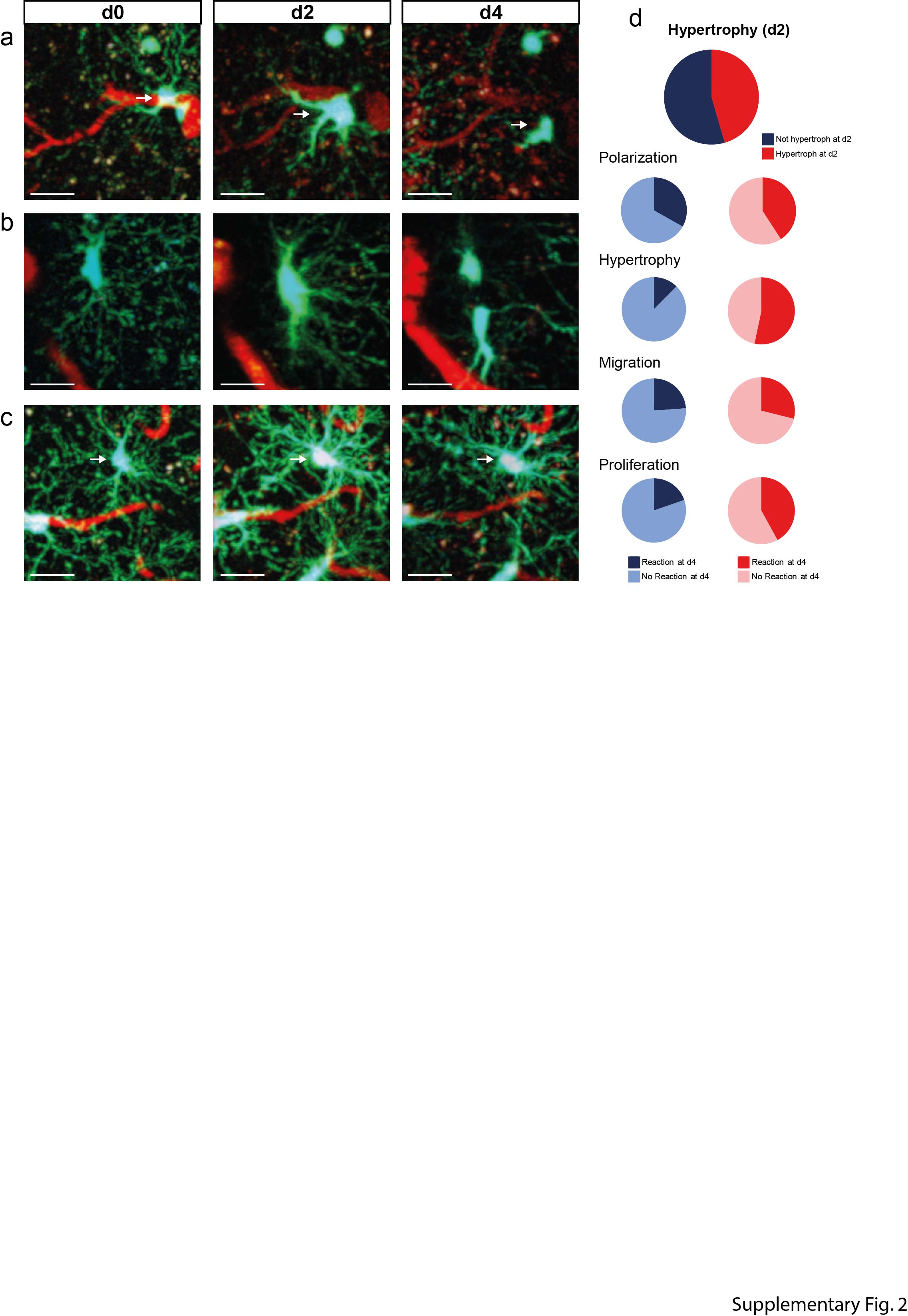

Supplement: Supplementary file 3 [file Image_2.JPEG]

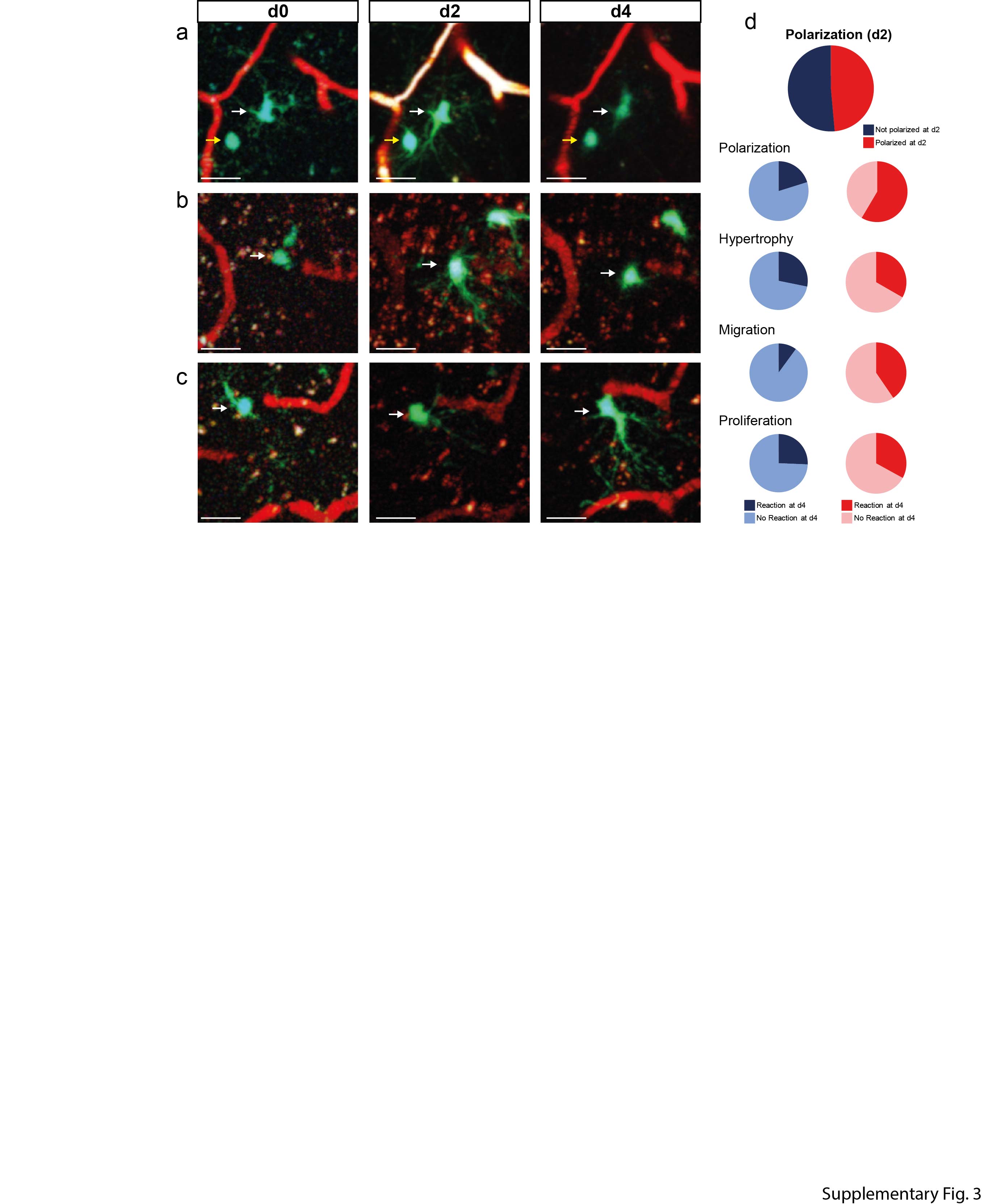

Supplement: Supplementary file 4 [file Image_3.JPEG]

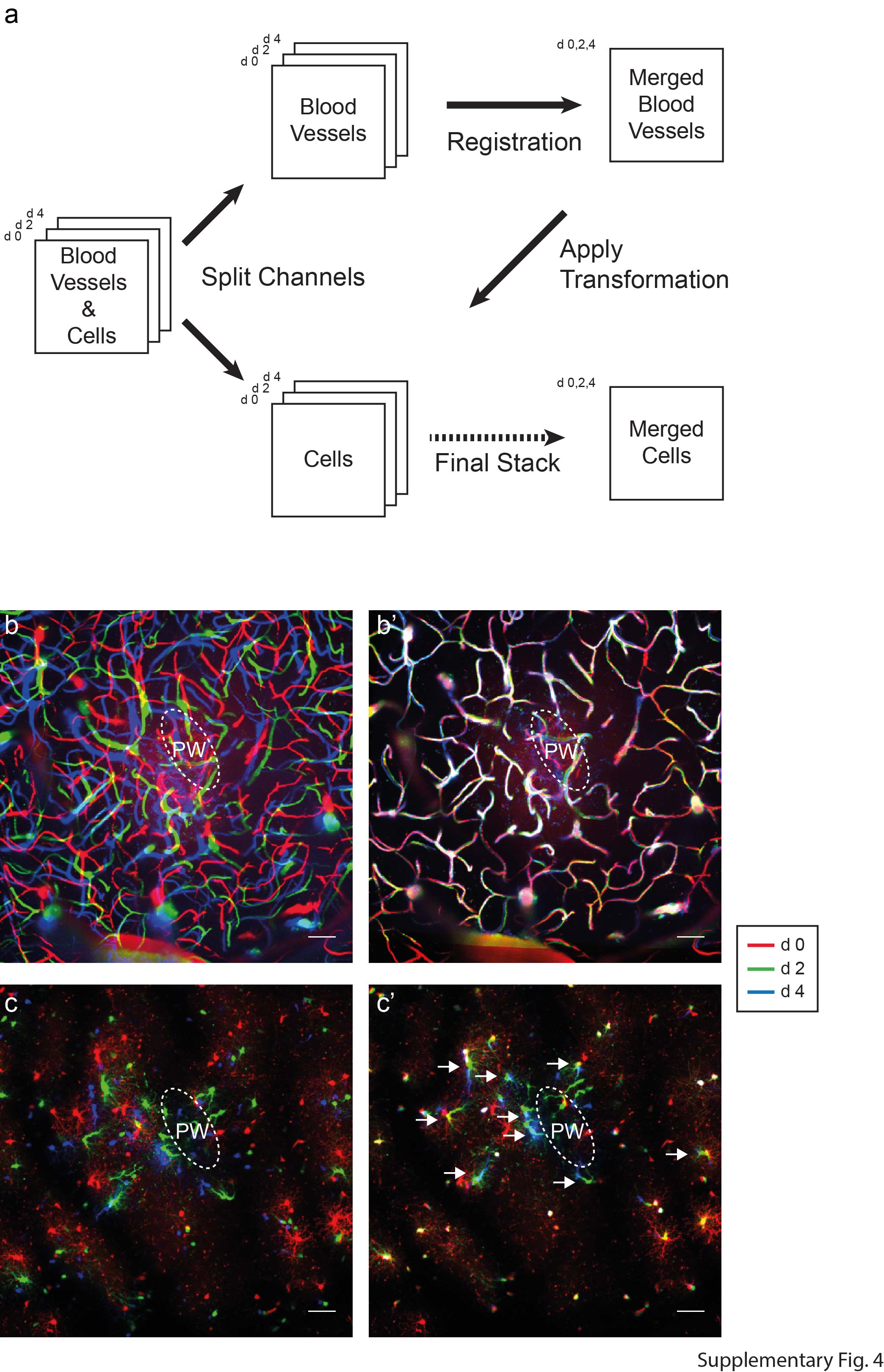

Supplement: Supplementary file 5 [file Image_4.JPEG]

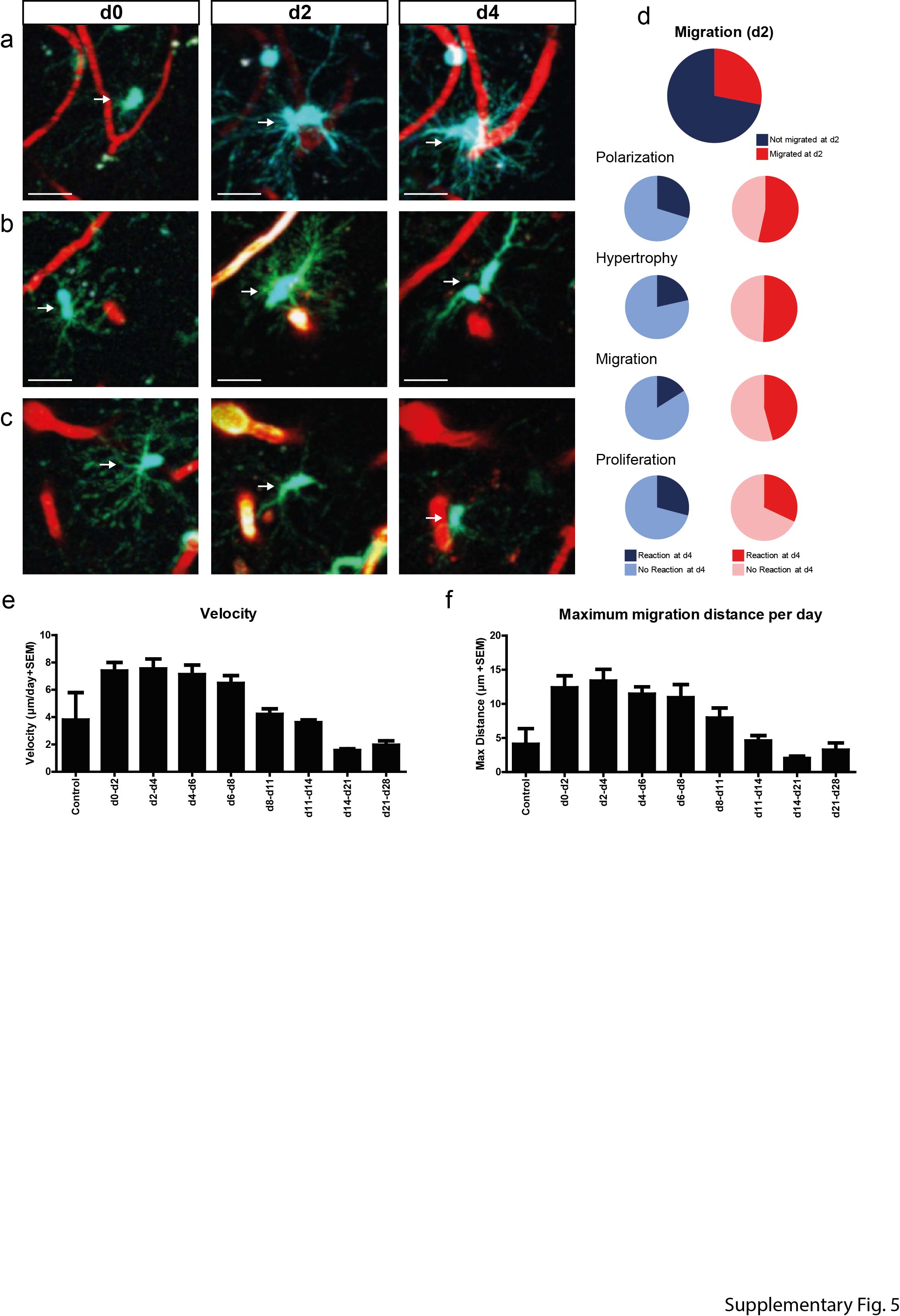

Supplement: Supplementary file 6 [file Image_5.JPEG]

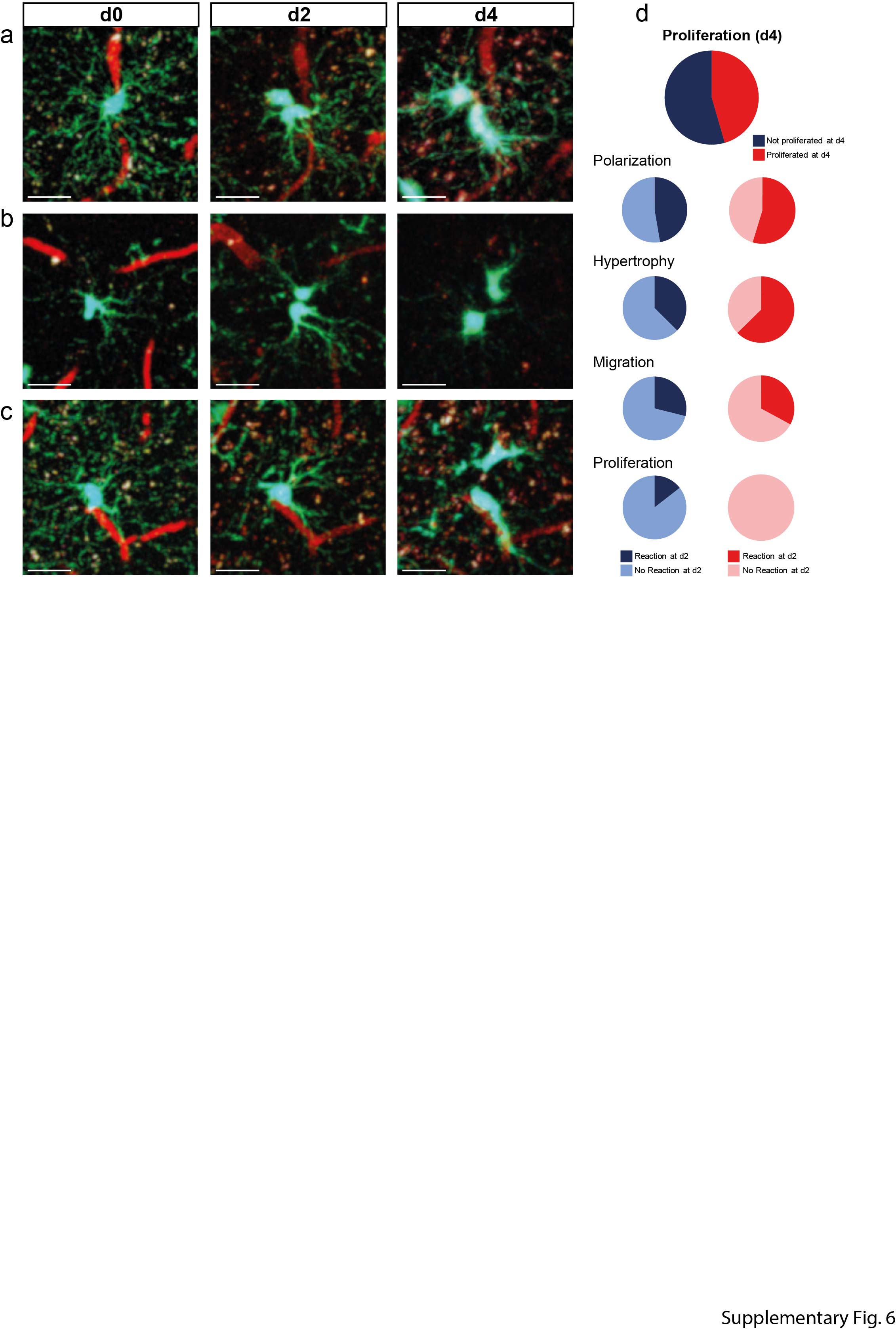

Supplement: Supplementary file 7 [file Image_6.JPEG]
